# Management of Endometrial Hyperplasia: A Comparative Review of Guidelines

**DOI:** 10.3390/cancers17193143

**Published:** 2025-09-27

**Authors:** Eirini Boureka, Ioannis Tsakiridis, Georgios Kapetanios, Georgios Michos, Sonia Giouleka, Anastasios Liberis, Apostolos Mamopoulos, Themistoklis Dagklis, Ioannis Kalogiannidis

**Affiliations:** Third Department of Obstetrics and Gynecology, School of Medicine, Faculty of Health Sciences, Aristotle University of Thessaloniki, 54642 Thessaloniki, Greece; ebourek@auth.gr (E.B.); kapetaniosg@hotmail.gr (G.K.); giomichos@hotmail.com (G.M.); gkiouleka@auth.gr (S.G.); alymperi@auth.gr (A.L.); amamop@auth.gr (A.M.); dagklis@auth.gr (T.D.); ikalogia@auth.gr (I.K.)

**Keywords:** endometrial hyperplasia, hyperplasia without atypia, atypical hyperplasia, hysteroscopy, management, guidelines, comparison

## Abstract

**Simple Summary:**

Endometrial hyperplasia, with or without atypia, is considered a precursor of cancer, affecting a great number of women around the world. This study aimed to compare influential guidelines on the management of this clinical entity. Endometrial hyperplasia without atypia is mainly managed conservatively, with observation of medical treatment, reserving hysterectomy for specific cases. Atypical hyperplasia is managed primarily surgically, except in women desiring fertility or who are unfit for surgery. Regular surveillance with biopsies and long-term follow-up is universally recommended, but diagnostic and treatment approaches vary among the reviewed medical societies.

**Abstract:**

Endometrial hyperplasia, presenting without atypia (EH) or as atypical hyperplasia (AH), is considered a precursor of endometrial cancer and affects women of reproductive or perimenopausal age, posing a major public health concern. The aim of this study was to review and compare the most recently published influential guidelines providing recommendations on the management of endometrial hyperplasia. Thus, a comparative review of guidelines from the Royal College of Obstetricians and Gynecologists, the Society of Obstetricians and Gynecologists of Canada, and the American College of Obstetricians and Gynecologists was conducted. There is a consensus regarding the optimal management strategies for EH, with observation and medical treatment being the first-line options and surgical treatment with total hysterectomy offering a second line in specific cases. Moreover, there is agreement regarding patients with AH, with surgical treatment being the recommended approach, while medical therapy is preferred for women who seek fertility preservation. Notably, close surveillance with endometrial biopsies every 3 or 6 months is suggested unanimously, as well as long-term follow-up in high-risk patients. Controversy exists regarding the initial diagnostic approach, with RCOG and SOGC suggesting outpatient endometrial biopsy, while ACOG recommends diagnostic hysteroscopy, as well as the therapeutic regimens for the oral treatment of EH. Surgical techniques such as endometrial ablation, intraoperative frozen section analysis, intraoperative visual inspection of the uterus, and morcellation constitute areas of controversy among the reviewed guidelines, and the surveillance protocols for women with EH are addressed differently between RCOG and SOGC. Notably, RCOG is the only medical society offering recommendations regarding women under HRT and those on therapy for breast cancer. The development of consistent international practice protocols for timely management strategies and surveillance protocols is of paramount importance to safely guide clinical practice and subsequently improve women’s health.

## 1. Introduction

Endometrial hyperplasia refers to the abnormal proliferation of the endometrial glands, which results in an increased gland-to-stroma ratio compared to a normal proliferative endometrium [1]. Its incidence is reported approximately at 133 per 10,000 women [2], and it behaves as a precursor lesion of endometrial cancer, with almost one-third of women with hyperplasia eventually diagnosed with endometrial cancer [3]. According to the revised 2014 World Health Organization (WHO) classification, hyperplasia is divided into two groups based on the presence or absence of atypia: endometrial hyperplasia without atypia (EH) and atypical hyperplasia (AH) [4].

The most prevalent risk factor for the development of endometrial hyperplasia is the prolonged unopposed exposure to estrogens; this includes women with obesity, with a nearly 23-fold higher risk of hyperplasia [5], as well as those with polycystic ovarian syndrome (PCOS), estrogen-secreting ovarian tumors, and nulliparous or postmenopausal women [6]. Moreover, the risk is elevated in cases with exogenous estrogen intake, such as those undergoing hormonal replacement therapy (HRT) or breast cancer patients on long-term tamoxifen therapy [7].

Hyperplasia commonly presents with abnormal uterine bleeding, which may involve changes in the duration, frequency, or quantity of menstrual bleeding, as well as the presence of intermenstrual bleeding [8]. In postmenopausal women, any type of vaginal bleeding is considered abnormal and requires investigation for endometrial hyperplasia/cancer [9]. Transvaginal ultrasound is often used to aid the diagnostic procedure [10], while the gold standard for diagnosing endometrial hyperplasia/cancer is histological examination of the endometrial tissue [11].

According to the histological classification, the management of endometrial hyperplasia may be surgical or conservative, with total hysterectomy recommended in cases that are atypical, while medical therapy is recommended in cases of EH [12]. However, this approach is not universally applicable across all patient populations, as factors related to age, fertility desire, comorbidities, and patient preferences must be carefully considered in the treatment planning process. These facts underline the importance of individualized management strategies based on the development and implementation of uniform international evidence-based algorithms to ensure optimal care and prevent hyperplasia progression to endometrial cancer. Thus, the aim of this descriptive review was to summarize and compare the most recent recommendations from influential guidelines on the management of endometrial hyperplasia.

### 1.1. Evidence Acquisition

The most recently published guidelines on the management of endometrial hyperplasia were retrieved, and a descriptive review was conducted. In particular, three guidelines were identified from the Royal College of Obstetricians and Gynecologists (RCOG 2016) [13], the Society of Obstetricians and Gynecologists of Canada (SOGC 2019) [14], and the American College of Obstetricians and Gynecologists (ACOG 2023) [15].

All three medical societies’ literature was searched in the Cochrane Library (RCOG, SOGC, ACOG), PubMed (SOGC, ACOG), EMBASE, MEDLINE (RCOG, ACOG), and CINAHL (RCOG), combining terms for endometrial hyperplasia and treatment (medical, surgery, progestin therapy, LNG-IUS, intrauterine devices, conservative therapy). Studies were limited to those in humans and English language, and the literature search was conducted for studies published until 2015 (RCOG), from 2000 to 2018 (SOGC) and from 2000 to 2021 (ACOG).

An overview of recommendations is presented in Table 1 (EH) and Table 2 (AH).

The corresponding levels of evidence are presented in Table 1 and Table 2. Notably, ACOG states that each recommendation was developed following approval by at least 75% of voting members.

### 1.2. Diagnosis of Endometrial Hyperplasia

All the reviewed guidelines agree that the diagnosis of endometrial hyperplasia is based on the histological examination of an endometrial biopsy. However, discrepancies exist regarding the optimal diagnostic method, as RCOG and SOGC primarily support outpatient endometrial tissue sampling, such as Pipelle biopsy, while ACOG recommends diagnostic hysteroscopy. RCOG and SOGC make their recommendation based on a systematic review showing that the pooled likelihood ratio of a positive result was 12.0 (95% CI: 7.8–18.6) when diagnosing hyperplasia, while that of a negative result was 0.2 (95% CI: 0.1–0.3) [16]. Moreover, the feasibility and convenience of this method are underlined by RCOG and SOGC. On the other hand, ACOG is against this diagnostic method based on studies to have shown that in patients undergoing hysterectomy, a higher rate of endometrial cancer was identified in those initially evaluated with Pipelle biopsy compared to those assessed by dilation and curettage (D&C) [17,18]. Additionally, ACOG recommends diagnostic hysteroscopy based on studies that underline its superiority over both the other techniques. More specifically, hysteroscopy demonstrated a significantly higher sensitivity compared to outpatient endometrial sampling (97.4% vs. 64.6%) [19]. Similarly, more cases of endometrial cancer were missed with D&C than with hysteroscopy [20]. Notably, both RCOG and SOGC point out the utility of diagnostic hysteroscopy, especially in cases of inconclusive results or negative results and ongoing abnormal uterine bleeding.

Additionally, RCOG mentions transvaginal ultrasound as a supplementary tool in the investigation of endometrial hyperplasia, as according to a meta-analysis, in patients with abnormal vaginal bleeding, visualization of the endometrium could reveal abnormalities, such as endometrial thickness, strengthening the indication for a biopsy [10].

Of note, the same diagnostic methods suggested for the initial diagnosis of endometrial hyperplasia are also recommended for surveillance. Histological evaluation remains necessary to ensure regression of the disease and detect any potential progression to endometrial cancer.

## 2. Management of Hyperplasia Without Atypia

### 2.1. Observation/Lifestyle Changes

RCOG and SOGC agree on the optimal management in patients with EH, while ACOG does not refer to this topic. In detail, RCOG and SOGC suggest that the initial approach may involve observation. However, medical management is required in cases where abnormal vaginal bleeding is present or if there is no regression of hyperplasia during the observation period. This is based on published data showing that EH carries a relatively low risk, around 5%, of progressing to endometrial cancer over the next 20-year period [21]. Additionally, results from two studies support a conservative approach with observation, demonstrating regression rates of 74% and 81% in women with EH without treatment, during a 6-month and 13-year follow-up period, respectively [22,23].

Notably, RCOG and SOGC highlight the importance of lifestyle modifications to reverse possible risk factors, such as a high body mass index (BMI). Evidence indicates that patients express a high acceptance of referral to bariatric specialists, especially when offered by gynecologic oncologists [24], contributing to a reduction in the long-term risk of endometrial hyperplasia [25].

However, in cases where observation results in disease progression, persistence of EH, or continued vaginal bleeding, medical treatment is proposed as the safest therapeutic option. According to small observational studies, administration of progestogens results in high regression rates, of up to 96%, compared to only 81% following observation [23,26]; therefore, when there is a suspicion or a high risk of progressive disease, treatment is essential.

### 2.2. Medical Management

RCOG and SOGC both agree that progestogens are the optimal choice for the medical treatment of EH. Both medical societies are in favor of administering local intrauterine progestogens [levonorgestrel-releasing intrauterine system (LNG-IUS)] as the first-line treatment and continuous oral (RCOG, SOGC) or injectable (SOGC) progestogens as the second choice. This is based on studies that highlighted the increased local release of levonogestrel on the endometrium, compared to oral progestogens, achieving therefore a higher response [27]. This is also underlined by a meta-analysis showing that LNG-IUS had a two-times higher regression rate (OR: 2.30; 95% CI: 1.39–3.82), compared to oral progestogens, after 3 months of therapy, while it presented with an almost eight-times higher regression rate in 24 months (OR: 7.46; 95% CI: 2.55–21.78) [28]. Moreover, the use of an intrauterine device promotes patient compliance and is associated with fewer systematic adverse effects, as systemic progestogen exposure is minimized [27], justifying the choice of LNG-IUS as the first-line treatment.

Regarding regimens, RCOG and SOGC make recommendations, with certain differences; medroxyprogesterone is recommended unanimously, although only RCOG mentions specific doses (10–20 mg/day). Moreover, norethisterone (RCOG, SOGC) at doses of 10–15 mg/day, as mentioned by RCOG, and aromatase inhibitors, such as letrozole or megestrole acetate, are suggested as alternatives. These recommendations are based on a study that showed that these regimens are most commonly used on EH and achieve a regression rate of approximately 66%, which, although significantly lower than LNG-IUS, is acceptable as a second-line treatment [29]. Interestingly, SOGC is the only medical society suggesting the administration of injectable medroxyprogesterone acetate as an acceptable alternative. This suggestion is based on a study showing that its regression rate reached 92% after 6 months of administration [30]. Notably, RCOG mentions that cyclical progestogens are not recommended due to studies showing that oral progestogens for 10 or 12 days per cycle achieved a lower regression rate compared to continuous administration [31,32,33]. On the other hand, SOGC proposes cyclic progestin intake as an acceptable alternative, without providing further justification.

Regarding the duration of the treatment, there is an overall agreement that LNG-IUS should be left in place for at least 6 months and up to 5 years, whereas oral or injectable treatment should be administered for 6 months. The 6-month cut-off for both local and systematic oral progestin administration is justified by a prospective study showing that, when the duration of therapy increased from 3 to 6 months, the regression rate improved significantly [34]. In detail, in the group treated with LNG-IUS, rates improved from 84% to 100%, while, in patients receiving continuous oral medroxyprogesterone, rates improved from 50% to 64% [34]. Moreover, results from a randomized study showed that, among women treated for EH either with LNG-IUS or with oral progestogens, almost one out of three relapsed after discontinuation of treatment, following the initial 6-month period [33]. Additionally, a meta-analysis of 24 observational studies identified it as a common practice to leave the local device in place for up to 5 years; this approach is supported by the device’s minimal adverse effects and its significantly greater benefits in preventing recurrence [26]. On the other hand, continuous administration of high doses of oral progestins is not supported, as there is a lack of well-designed studies to evaluate their long-term safety profile.

### 2.3. Surgical Management

Regarding surgical treatment of EH, both RCOG and SOGC unanimously agree that it should not be used as the first-line treatment; rather, it should be suggested only in individual cases. This assumes that progestogen therapy achieves disease regression in most cases, avoiding therefore any possible complications from a major surgery. However, if there is progression to AH or endometrial cancer, failure of medication to achieve regression within 12 months, persistent vaginal bleeding, or the patient declines medical treatment or follow-up, surgical treatment is an acceptable option. Results from a cohort study showed that in women whose initial treatment with LNG-IUS failed to achieve remission, hysterectomy was performed, resulting in endometrial cancer diagnosis in 33% of cases [35]. Moreover, in women who decline long-term progestogen treatment, or in whom hormone replacement is contraindicated, hysterectomy should be offered. The same applies to women for whom long-term follow-up is not feasible or who prefer a more radical approach for psychological reasons.

The recommended surgery is total hysterectomy with salpingo-oophorectomy in postmenopausal and salpingectomy in premenopausal women. Although data on the outcomes of subtotal hysterectomy in women with EH are limited, retention of the cervix is not recommended, as hyperplasia may extend into the endocervix. Additionally, given the fact that high-grade serous ovarian cancer may originate in the fallopian tube, the suggestion of salpingo-oophorectomy in postmenopausal and salpingectomy in premenopausal women seems reasonable [36]. Moreover, in premenopausal women, oophorectomy may be performed based on the individual’s risk of ovarian cancer and patient preferences. Of note, the laparoscopic approach is generally preferred due to its shorter recovery time and lower need for postoperative analgesia [37]; however, SOGC supports either a vaginal, abdominal, or laparoscopic approach given the fact that EH is a non-cancerous condition.

Controversy exists regarding the performance of endometrial ablation as a therapeutic technique, with RCOG stating that it is against it overall, while SOGC suggests that it may be considered in cases where surgery is contraindicated. RCOG bases its recommendation on a study showing that of 16 cases with hyperplasia treated with endometrial ablation, one progressed to endometrial cancer, demonstrating a lack of complete endometrial tissue destruction [38]. Moreover, RCOG underlines that any possible adhesions created would complicate adequate follow-up. SOGC agrees with RCOG regarding the difficulties in follow-up and the possible incomplete destruction of the endometrium. However, this medical society supports ablation as a safe alternative in patients with EH based on a large prospective study showing that from 161 cases of EH, only 12 underwent hysterectomy after endometrial ablation, and no cases with residual hyperplasia were identified [39].

### 2.4. Follow-Up

Regarding surveillance for EH, RCOG and SOGC when referring to the topic recommend different strategies; RCOG recommends at least two consecutive negative biopsies at 6-month intervals before discharging the patient from follow-up, while for patients at a high risk of recurrence, long-term annual surveillance should be considered. As for the method, similarly to the initial diagnostic strategy, RCOG supports outpatient endometrial biopsy initially and hysteroscopy as an alternative. According to published data, in more than half of the patients who underwent hysterectomy after initial treatment for endometrial hyperplasia, evidence of relapse of the disease was detected in the final specimen [40]. Moreover, given the lack of large-scale randomized trials to guide a safe biopsy interval, it is reasonable to follow a 6-month period for endometrial sampling, based on observational studies’ protocols [40,41]. Additionally, results from a cohort study showed a statistically significant association of hyperplasia relapse in a 9-year follow-up period in women with a BMI over 35 (HR: 18.93; 95% CI: 3.93–91.15) [41]. Therefore, it seems reasonable to adopt a long-term surveillance strategy in patients with persistent risk factors, such as a high BMI, to avoid potential future cancer cases.

SOGC, on the other hand, suggests that the first biopsy should be performed mid-therapy, approximately at 3 months after initiation of oral or injectable treatment and a second one 3 weeks after the completion of therapy. However, in case of treatment with LNG-IUS, SOGC suggests surveillance with endometrial biopsy every 3 to 6 months, with the device left in place. This is based on studies showing that the mean follow-up interval was 3 to 6 months to assess the endometrial response to treatment [26,42]. Moreover, diagnostic hysteroscopy with the LNG-IUS in place is supported based on a pilot study that followed this strategy with no reported complications [43]. Additionally, SOGC, in agreement with RCOG, proposes the extension of follow-up in cases of women with persistent risk factors.

## 3. Management of Atypical Hyperplasia

### 3.1. Surgical Management

Regarding AH, relevant recommendations are provided by all three reviewed medical societies. More specifically, there is an overall agreement in relation to the optimal management strategy in patients presenting with AH, with all guidelines recommending total laparoscopic hysterectomy as the first choice. This is based on a meta-analysis showing that the prevalence of concurrent endometrial cancer in patients with AH was high, reaching 32.6% (95% CI: 24.1–42.4%). Additionally, the overall risk of progression to cancer was also high, with an annual incidence rate of 8.2% (95% CI: 3.9–17.3%) [3]. Therefore, the recommendation to proceed to total hysterectomy is justified, to treat undiagnosed cases and prevent progression to endometrial cancer in the rest. Moreover, similarly to EH, retaining the cervix is contraindicated unanimously, as the lower endometrial segment might not be completely removed, allowing possible progression to invasive endometrial cancer. Of note, supporting the same strategy in AH as in EH, bilateral salpingo-oophorectomy in postmenopausal and salpingectomy with or without oophorectomy in premenopausal women are supported surgical strategies by all three medical societies.

Discrepancies exist regarding the use of intraoperative frozen section analysis during hysterectomy, with RCOG and SOGC stating they are against it, while ACOG suggests that it could be useful in settings where a gynecologic oncologist is available [44]. RCOG and SOGC base their recommendation on a retrospective study showing that the final histological examination differed from the results of the frozen section analysis in more than 60% of cases; in 57% of patients, the frozen section analysis failed to diagnose endometrial cancer, which was revealed later in the final histology [45]. On the other hand, according to another study, 98.6% of cases of endometrial cancer were diagnosed by frozen section analysis, and from the ones missed, only 1 out of 14 had myometrial invasion [46]. As a result, ACOG supports the use of this intraoperative technique, in settings where a gynecologic oncologist is available and can manage any potential cancer diagnosis, accordingly. Interestingly, according to a cost-effectiveness analysis, when hysterectomies for endometrial cancer were performed by expert gynecologic oncologists, the overall benefit reached USD 116 million and 180 quality-adjusted life years, highlighting the significant impact of surgical expertise and specialization in malignant procedures [47].

In addition to surgical procedures, visual inspection of the endometrial cavity post-surgery is not recommended by SOGC due to a lack of adequate data to support this technique. Rather, the existent studies appear conflicting, either supporting that gross examination can correctly diagnose the endometrial cancer stage [48], or stating that it leads to unnecessary procedures and overtreatment [49]. Therefore, due to insufficient justification for this procedure and the potential interference that uterine intraoperative examination might cause to the histological examination, SOGC is against its routine use. However, similarly to frozen section analysis, ACOG supports the consideration of visual endometrial examination, to evaluate myometrial invasion, only in cases where it can be managed appropriately by a specialized surgeon.

Moreover, routine lymphadenectomy is contraindicated by RCOG and SOGC as, despite the high incidence (almost 43%) of concurrent endometrial carcinoma in AH patients [50], routine systematic pelvic lymphadenectomy is associated with more postoperative complications and insignificant overall and disease-free survival [51]. More specifically, results from a randomized trial showed that pelvic lymphadenectomy failed to benefit overall survival (HR: 1.04; 95% CI: 0.74–1.45) and recurrence-free survival (HR: 1.25; 95% CI: 0.93–1.66) [52].

Similarly, the morcellation of the uterus is not recommended by RCOG and SOGC as potential cancerous cells may spill into the abdominal cavity. However, ACOG supports the use of this procedure when necessary, highlighting that it should be performed in a contained environment, such as a bag, to avoid any potential leak. Additionally, the difficulty of a proper histological examination of the specimen should be taken into account, prior to the decision to use a morcellator.

Of note, endometrial ablation is a matter of controversy among the reviewed guidelines. In detail, RCOG and ACOG state they are against it, while SOGC suggests that it can be of use in patients who cannot undergo surgery. As previously mentioned in patients with EH, endometrial ablation cannot guarantee complete endometrial destruction [38]. Moreover, given the high prevalence of concurrent endometrial carcinoma in AH patients, the risk of remaining endometrial tissue is significantly high. SOGC, on the other hand, while recognizing the potential risk, mentions that in patients with comorbidities, who are unfit for surgery, it can be considered in collaboration with gynecologic oncologists.

### 3.2. Medical Management

Contrary to what applies in EH, medical treatment in AH is considered an alternative only in women who wish to preserve their fertility or cannot undergo major surgery due to other underlying health conditions. There is agreement on the first choice of treatment, with all three medical societies supporting the use of LNG-IUS. This is supported by a multicenter randomized study showing that in women with endometrial hyperplasia, treatment with LNG-IUS resulted in a 100% response, compared to 96% in those receiving oral progestogens [33]. Moreover, given the fewer adverse events related to the intrauterine device, it is considered as the most suitable option for medical treatment [53]. Notably, patients are more likely to comply with this specific regimen not only because of its feasibility but also due to its association with less weight gain compared to oral progestogen administration [54].

As a second-line treatment, oral progestogens (RCOG, SOGC, ACOG), aromatase inhibitors, GnRH agonists (SOGC), or a combination of LNG-IUS and oral progestogens (ACOG) are suggested. Several studies demonstrate an acceptable regression rate in women with AH receiving these regimens [55,56,57]. However, most of those women underwent medical treatment primarily for fertility-sparing purposes and were strongly advised to remain under close surveillance. Moreover, in case of disease progression, failure to achieve regression, or completion of childbearing, proceeding to definitive surgical treatment was highly recommended. Additionally, SOGC mentions the administration of metformin supplementary to progestogens, as studies show it has the potential to increase the overall treatment effect [58,59].

Of note, ACOG suggests long-term maintenance therapy if persistent risk factors are present. This is justified by a study showing that when risk factors remain after the first year of treatment of AH, the overall 20-year progression risk increased from 8.2% (95% CI: 1.3–14.6%) to 27.5% (95% CI: 8.6–42.5%) [21].

### 3.3. Fertility Sparing

Fertility sparing is a matter discussed by all reviewed guidelines. More specifically, all three medical societies agree on the importance of careful counseling about the potential risks of progression to endometrial cancer and invasive disease. For that reason, detailed investigation is necessary to rule out undiagnosed cancer and provide the optimal advice and treatment strategy [60]. According to a meta-analysis, in women with AH who underwent fertility-sparing treatment, the risk of disease progression to endometrial cancer was 2%, while the risk of metastatic cancer or death was 0.5% [61]. Moreover, medical treatment is essential to avoid hysterectomy and preserve the uterus for future pregnancy. Data from a cohort study showed that in women with AH who received medical treatment with progestins, the overall risk of progression to cancer was significantly lower, at 0.15 (95% CI: 0.07–0.31), compared to those without treatment [62]. RCOG and SOGC point out that once fertility is no longer required or wished, hysterectomy is the optimal treatment plan to avoid future progression to endometrial cancer. Data from a study showed that the relapse rate was significantly high in patients with AH, at 27.3% [63].

With regards to the optimal strategy towards achieving a pregnancy, all three reviewed medical societies agree that assisted reproductive technology (ART) is preferred over spontaneous conception. This is justified by a study showing that the rate of live birth was higher in the ART group compared to the one with spontaneous conception (39.4% vs. 14.9%; *p* = 0.001, respectively) [61]. Moreover, data show that women who received treatment for in vitro fertilization did not present with a higher incidence of recurrence [64]. Additionally, during a 2-year period, about 20% of women achieving a pregnancy experienced recurrence, compared to a three-times higher rate of 62% of women not achieving pregnancy (*p* = 0.002; 95% CI: 0.06–0.61) [64]. This fact indicates that pregnancy might pose a protective factor in women with endometrial hyperplasia. Notably, RCOG points out that at least one negative biopsy is important before making decisions for a future pregnancy. This is important as data show that in women receiving treatment with LNG-IUS, the overall pregnancy rates were significantly higher compared to the ones without treatment (46.06% vs. 28.04%, respectively), possibly indicating that resolution of the hyperplastic endometrium contributed to a successful implantation [65].

### 3.4. Follow-Up

Regarding the optimal follow-up strategy for women with AH, all three guidelines agree they should undergo endometrial biopsy every three (RCOG, SOGC, ACOG) or six (ACOG) months, for at least two consecutive negative results (RCOG, SOGC) or up to two years (ACOG). Results from a systematic review showed that the median time until complete response was 6 months, with initial signs observed as early as 3 months [42]. Additionally, results from another study showed that in medically treated patients with AH, the median time to progression to cancer was 9.5 months (IQR: 3.5–28.6) [66]; therefore, the implementation of a more rigorous surveillance protocol would be justified to prevent future cancer cases. Notably, ACOG mentions specifically that surveillance is necessary only in patients with a preserved uterus, as hysterectomy is considered a definitive treatment in patients with AH [67]. Moreover, RCOG and SOGC suggest that long-term surveillance should consist of biopsies every 6–12 months until treatment with hysterectomy is performed. This is based on the findings of a multicenter randomized trial demonstrating that disease relapse occurred within a 24-month surveillance period, regardless of whether patients were treated with cyclic or continuous oral progestogens or LNG-IUS, with no significant difference among treatment modalities (*p* = 0.66) [68]. Furthermore, if treatment failure persists after one year, a discussion of alternative management options is strongly advised.

## 4. Management of Specific Conditions

### 4.1. Hyperplasia on Endometrial Polyp

Specific recommendations regarding the diagnosis of endometrial hyperplasia on a polyp are made by RCOG and SOGC and are in agreement. In detail, the recommended approach after the initial diagnosis of hyperplasia includes the complete evaluation of the entire endometrial cavity and treatment planning according to the histological classification. Results from a study showed that in 50% of women with EH and 57% of those with AH, hyperplasia was present in the surrounding endometrium [69]. Additionally, results from a systematic review showed that the pooled risk of concurrent cancer in women with atypical endometrial polyps was 5.6% (95% CI: 0.2–17.6%) [70].

### 4.2. Hormonal Replacement Therapy and Endometrial Hyperplasia

RCOG is the only medical society referring to HRT in relation to endometrial hyperplasia. More specifically, it mentions that systemic estrogen-only HRT is not recommended in women who have a uterus, as data from a Cochrane review showed that unopposed estrogen intake for up to 2 years was associated with a significantly high incidence of endometrial hyperplasia, even at low doses; low estrogen intake almost tripled the risk of EH in a year (OR: 2.84; 95% CI: 0.94–8.29), while the highest risk was documented for a 3-year moderate-dose estrogen intake, which increased the risk of hyperplasia by 16 times (OR: 15.99; 95% CI: 9.28–27.54) [71]. Additionally, women under sequential HRT should be advised to switch to continuous combined therapy, according to several studies that show a relative higher risk of hyperplasia in women who receive sequential HRT compared to continuous combined treatment [72,73]. Moreover, in these cases, remission of hyperplasia was documented after discontinuation of HRT or a switch to continuous combined therapy [73]. Of note, RCOG mentions that all women, including those on continuous combined therapy, should be reviewed regarding their wish to continue treatment.

### 4.3. Breast Cancer and Endometrial Hyperplasia

Regarding treatment for women with a history of breast cancer, RCOG (SOGC and ACOG make no relevant recommendations) mentions that they should be carefully informed that tamoxifen is related to an increased risk of endometrial hyperplasia, in contrast to aromatase inhibitors, which are not. Results from a study showed that tamoxifen intake for more than 2 years is associated with a two-times higher risk of endometrial cancer (RR: 2.3; 95% CI: 0.9–5.9) [74]. Moreover, results from a large study showed that this risk was significant only in women aged more than 50 years (RR: 5.33; 95% CI: 2.47–13.17) [75]. On the other hand, treatment with aromatase inhibitors was not associated with an increased risk of endometrial pathology or vaginal bleeding compared to tamoxifen intake (OR: 0.45; 95% CI: 0.16–1.32) [76]. Hence, in women with endometrial hyperplasia, reassessment of the need for tamoxifen is advised by RCOG, as well as consultation with an experienced oncologist. Although robust evidence is lacking, the increased incidence of endometrial cancer among women receiving tamoxifen suggests that pre-existing hyperplasia may progress to carcinoma [74]. Therefore, careful evaluation of tamoxifen use in this patient group is essential, along with a multidisciplinary approach to management. Notably, as the long-term effect of LNG-IUS in breast cancer patients is uncertain, it is not recommended in this patient group. Data from two studies showed that the use of endometrial progestogen devices in women on tamoxifen led to a reduced incidence of endometrial polyps (Peto OR: 0.14; 95% CI: 0.03–0.61) [77] and endometrial hyperplasia (OR: 0.13; 95% CI: 0.03–0.58) [78]. Additionally, several studies showed that it is not significantly associated with an increased risk of breast cancer or death [79,80]. However, due to the limitations of the existing studies and the uncertainty regarding long-term effects on breast cancer recurrence, it is generally advised to avoid its use.

## 5. Conclusions

To summarize, this review shows that there is a consensus among the reviewed guidelines regarding the optimal management strategies for EH, with observation and medical treatment, primarily with LNG-IUS and secondly with oral progestogens, offering safe alternatives. Moreover, surgical treatment with total laparoscopic hysterectomy is considered a second-line option in individuals with EH and should be accompanied with salpingectomy in premenopausal, or salpingo-oophorectomy in postmenopausal women. Regarding AH, all medical societies agree that total hysterectomy is the treatment of choice, but routine pelvic lymphadenectomy should be avoided. Moreover, there is agreement in relation to the indications of medical treatment, with all guidelines suggesting it should be used in patients unsuitable for major surgery or those wishing for fertility preservation, and that ART appears advantageous compared to spontaneous conception. Notably, close surveillance with endometrial biopsies every 3 or 6 months is suggested unanimously, as well as long-term follow-up in high-risk patients.

Several discrepancies are detected in relation to the initial diagnostic approach, with RCOG and SOGC suggesting outpatient endometrial biopsy, while ACOG favors diagnostic hysteroscopy, as well as therapeutic regimens for the oral treatment of EH. Of note, techniques such as endometrial ablation, intraoperative frozen section analysis, intraoperative visual inspection of the uterus, and morcellation are areas of controversy among the reviewed guidelines. Moreover, the follow-up strategy for women with EH is a matter addressed differently between RCOG and SOGC. Notably, RCOG is the only medical society making specific recommendations regarding women under HRT and those on tamoxifen therapy for breast cancer treatment.

To conclude, endometrial hyperplasia presents as a significant precursor for endometrial cancer, highlighting the importance of early detection and appropriate management to prevent malignant progression. Therefore, timely management strategies and close surveillance protocols guided by evidence-based algorithms are essential to improve health outcomes and reduce mortality, especially in high-risk populations. Thus, the implementation of consistent international practice protocols is of the utmost importance to ensure standardized care and optimize women’s health.

## Figures and Tables

**Table 1:** Recommendations on hyperplasia without atypia.

	RCOG	SOGC	ACOG
**Country**	United Kingdom	Canada	United States
**Issued**	2016	2019	2023
**Title**	Management of Endometrial Hyperplasia	Guideline No. 390-Classification and Management of Endometrial Hyperplasia	Management of Endometrial Intraepithelial Neoplasia or Atypical Endometrial Hyperplasia
**Pages**	31	12	10
**References**	99	79	64
**Diagnostic method**	Outpatient endometrial tissue sampling (Level 2++) Diagnostic hysteroscopy Transvaginal ultrasound (Level 2++)	Outpatient endometrial tissue sampling (strong, high grade) Diagnostic hysteroscopy	Diagnostic hysteroscopy
**Hyperplasia on polyp**	Sampling of background endometrium recommended (Level 3) Management based on histology	Sampling of background endometrium recommended (low grade) Management based on histology	Not mentioned
**Management**	→Observation or (Level 2++) If observation fails or abnormal bleeding →Medical treatment (Level 2+)	→Observation or If observation fails or abnormal bleeding →Medical treatment (weak, low grade)	Not mentioned
**Medical treatment**	1st line: LNG-IUS (at least 6 months, up to 5 years) 2nd line: continuous oral progestogens (6 months) (Level 1+)	1st line: local intrauterine progestogens (at least 6 months, up to 5 years) 2nd line: continuous oral or injectable progestogens (6 months) (strong, high grade)	Not mentioned
**Regimens**	-Medroxyprogesterone 10–20 mg/day -Norethisterone 10–15 mg/day Cyclical progestogens not recommended (Level 1+)	-Injectable medroxyprogesterone -Aromatase inhibitors (letrozole, megestrole acetate) -Norethisterone	Not mentioned
**Hysterectomy**	Not considered as first-line treatment (Level 2+) Laparoscopic approach preferred (Level 1+) Individualization In postmenopausal women, offer bilateral salpingo-oophorectomy In premenopausal women, offer salpingectomy Subtotal hysterectomy not recommended (Level 4)	Not considered as first-line treatment Individualization In postmenopausal women, offer bilateral salpingo-oophorectomy In premenopausal women, offer salpingectomy (moderate grade) Subtotal hysterectomy not recommended (strong, low grade)	Not mentioned
**Endometrial ablation**	Not recommended (Level 1−)	Consider if unfit for surgery (low grade)	Not mentioned
**Follow-up**	→Outpatient endometrial biopsy (Level 2++) →Hysteroscopy, as an alternative At least 2 consecutive 6-monthly negative biopsies before discharge At high risk: long-term annual follow-up (Level 2+)	Oral/injectable regimens: -1st biopsy at 3 months -2nd biopsy 3 weeks after completion of treatment (strong, very low grade) LNG-IUS: Biopsy every 3–6 months At high risk: long-term follow-up	Not mentioned

LNG-IUS: Levonorgestrel-releasing intrauterine system.

**Table 2:** Recommendations on atypical hyperplasia.

	RCOG	SOGC	ACOG
**Management**	Total laparoscopic hysterectomy (Level 1+) Postmenopausal women: + bilateral salpingo-oophorectomy (good practice point) Premenopausal women: + salpingectomy +/− oophorectomy (Level 2++) Subtotal hysterectomy not recommended (Level 4)	Total laparoscopic hysterectomy Postmenopausal women: + bilateral salpingo-oophorectomy (strong, moderate) Premenopausal women: + salpingectomy +/− oophorectomy (low grade) Subtotal hysterectomy not recommended (strong, low grade)	Total laparoscopic hysterectomy Postmenopausal women: + bilateral salpingo-oophorectomy Premenopausal women: + salpingectomy +/− oophorectomy Subtotal hysterectomy not recommended
**Intraoperative frozen section analysis**	Not recommended (Level 2+)	Not recommended (low grade)	Should be considered only in places where gynecologic oncologist is available
**Visual inspection**	Not mentioned	Not recommended	Should be considered only in places where gynecologic oncologist is available
**Lymphadenectomy**	Not recommended (Level 2++)	Not recommended (moderate grade)	Not mentioned
**Morcellation**	Should be avoided (Level 4)	Not recommended	If needed, it should be performed in a contained environment
**Endometrial ablation**	Not recommended (Level 2+)	Consider if unfit for surgery Consult gynecology oncology	Not recommended
**Medical treatment**	1st line: LNG-IUS 2nd line: oral progestogens (Level 2++)	1st line: LNG-IUS 2nd line: oral progestogens aromatase inhibitors GnRH agonists + Metformin	1st line: LNG-IUS 2nd line: oral progestogens Long-term maintenance therapy if persistent risk factors
**Fertility sparing**	→Careful counseling →Careful investigation →Medical treatment (Level 4) Once fertility is not required, proceed to hysterectomy (Level 2++)	→Careful counseling →Careful investigation →Medical treatment Once fertility is not required, proceed to hysterectomy	→Careful counseling →Careful investigation →Medical treatment
**Prenatal plan**	At least one negative biopsy (Level 4) Consider ART over natural conception (Level 2++)	Consider ART over natural conception	Consider ART over natural conception
**Follow-up**	Endometrial biopsy every 3 months (2 consecutive negative results) (Level 4) Long-term follow-up → biopsy every 6–12 months until hysterectomy (Level 2++)	Endometrial biopsy every 3 months (2 consecutive negative results) Long-term follow-up → biopsy every 6–12 months until hysterectomy	Endometrial biopsy every 3–6 months for up to 2 years If no response after 9–12 months, discuss other management options

LNG-IUS: Levonorgestrel-releasing intrauterine system, ART: Assisted reproductive technology.

## Data Availability

All the data used for this article are publicly available.

## References

[1] Young R. (2014). Monodermal Teratomas and Somatic-Type Tumours Arising from a Dermoid Cyst. WHO Classification of Tumours of Female Reproductive Organs.

[2] Reed S.D., Newton K.M., Clinton W.L., Epplein M., Garcia R., Allison K., Voigt L.F., Weiss N.S. (2009). Incidence of endometrial hyperplasia. Am. J. Obs. Obstet. Gynecol..

[3] Doherty M.T., Sanni O.B., Coleman H.G., Cardwell C.R., McCluggage W.G., Quinn D., Wylie J., McMenamin Ú.C. (2020). Concurrent and future risk of endometrial cancer in women with endometrial hyperplasia: A systematic review and meta-analysis. PLoS ONE.

[4] Emons G., Beckmann M.W., Schmidt D., Mallmann P., Uterus Commission of the Gynecological Oncology Working Group (2015). New WHO Classification of Endometrial Hyperplasias. Geburtshilfe Frauenheilkd..

[5] Epplein M., Reed S.D., Voigt L.F., Newton K.M., Holt V.L., Weiss N.S. (2008). Risk of complex and atypical endometrial hyperplasia in relation to anthropometric measures and reproductive history. Am. J. Epidemiol..

[6] Ricci E., Moroni S., Parazzini F., Surace M., Benzi G., Salerio B., Polverino G., Vecchia C.L.A. (2002). Risk factors for endometrial hyperplasia: Results from a case-control study. Int. J. Gynecol. Cancer.

[7] Chandra V., Kim J.J., Benbrook D.M., Dwivedi A., Rai R. (2016). Therapeutic options for management of endometrial hyperplasia. J. Gynecol. Oncol..

[8] Tsakiridis I., Giouleka S., Koutsouki G., Kostakis N., Kalogiannidis I., Kourtis A., Athanasiadis A., Goulis D.G., Dagklis T. (2022). Investigation and management of abnormal uterine bleeding in reproductive-aged women: A descriptive review of national and international recommendations. Eur. J. Contracept. Reprod. Health Care.

[9] Fraser I.S., Critchley H.O., Broder M., Munro M.G. (2011). The FIGO recommendations on terminologies and definitions for normal and abnormal uterine bleeding. Semin. Reprod. Med..

[10] Timmermans A., Opmeer B.C., Khan K.S., Bachmann L.M., Epstein E., Clark T.J., Gupta J.K., Bakour S.H., Bosch T.v.D., van Doorn H.C. (2010). Endometrial thickness measurement for detecting endometrial cancer in women with postmenopausal bleeding: A systematic review and meta-analysis. Obstet. Gynecol..

[11] Gimpelson R.J., Rappold H.O. (1988). A comparative study between panoramic hysteroscopy with directed biopsies and dilatation and curettage: A review of 276 cases. Am. J. Obstet. Gynecol..

[12] Gültekin M., Dogan N.U., Aksan G., Ozgul N. (2010). Management of endometrial hyperplasia. Minerva Ginecol..

[13] No G.-t.G. (2016). Management of endometrial hyperplasia. RCOG BSGE Jt. Guidel..

[14] Auclair M.H., Yong P.J., Salvador S., Thurston J., Colgan T.T.J., Sebastianelli A. (2019). Guideline No. 390-Classification and Management of Endometrial Hyperplasia. J. Obstet. Gynaecol. Can..

[15] (2023). Management of Endometrial Intraepithelial Neoplasia or Atypical Endometrial Hyperplasia: ACOG Clinical Consensus No. 5. Obstet. Gynecol..

[16] Clark T.J., Mann C.H., Shah N., Khan K.S., Song F., Gupta J.K. (2001). Accuracy of outpatient endometrial biopsy in the diagnosis of endometrial hyperplasia. Acta Obstet. Gynecol. Scand..

[17] Costales A.B., Schmeler K.M., Broaddus R., Soliman P.T., Westin S.N., Ramirez P.T., Frumovitz M. (2014). Clinically significant endometrial cancer risk following a diagnosis of complex atypical hyperplasia. Gynecol. Oncol..

[18] Suh-Burgmann E., Hung Y.-Y., Armstrong M.A. (2009). Complex atypical endometrial hyperplasia: The risk of unrecognized adenocarcinoma and value of preoperative dilation and curettage. Obstet. Gynecol..

[19] Dueholm M., Hjorth I.M.D., Dahl K., Ørtoft G. (2020). Hysteroscopic resectoscope-directed biopsies and outpatient endometrial sampling for assessment of tumor histology in women with endometrial cancer or atypical hyperplasia. Eur. J. Obstet. Gynecol. Reprod. Biol..

[20] Bedner R., Rzepka-Górska I. (2007). Hysteroscopy with directed biopsy versus dilatation and curettage for the diagnosis of endometrial hyperplasia and cancer in perimenopausal women. Eur. J. Gynaecol. Oncol..

[21] Lacey J.V., Sherman M.E., Rush B.B., Ronnett B.M., Ioffe O.B., Duggan M.A., Glass A.G., Richesson D.A., Chatterjee N., Langholz B. (2010). Absolute risk of endometrial carcinoma during 20-year follow-up among women with endometrial hyperplasia. J. Clin. Oncol..

[22] Kurman R.J., Kaminski P.F., Norris H.J. (1985). The behavior of endometrial hyperplasia. A long-term study of "untreated" hyperplasia in 170 patients. Cancer.

[23] Terakawa N., Kigawa J., Taketani Y., Yoshikawa H., Yajima A., Noda K., Okada H., Kato J., Yakushiji M., Tanizawa O. (1997). The behavior of endometrial hyperplasia: A prospective study. Endometrial Hyperplasia Study Group. J. Obstet. Gynaecol. Res..

[24] Jernigan A.M., Maurer K.A., Cooper K., Schauer P.R., Rose P.G., Michener C.M. (2015). Referring survivors of endometrial cancer and complex atypical hyperplasia to bariatric specialists: A prospective cohort study. Am. J. Obstet. Gynecol..

[25] Argenta P.A., Kassing M., Truskinovsky A.M., Svendsen C.A. (2013). Bariatric surgery and endometrial pathology in asymptomatic morbidly obese women: A prospective, pilot study. BJOG Int. J. Obstet. Gynaecol..

[26] Gallos I.D., Shehmar M., Thangaratinam S., Papapostolou T.K., Coomarasamy A., Gupta J.K. (2010). Oral progestogens vs levonorgestrel-releasing intrauterine system for endometrial hyperplasia: A systematic review and metaanalysis. Am. J. Obstet. Gynecol..

[27] Nilsson C.G., Haukkamaa M., Vierola H., Luukkainen T., Arcangeli P. (1982). Tissue concentrations of levonorgestrel in women using a levonorgestrel-releasing IUD. Clin. Endocrinol..

[28] Abu Hashim H., Ghayaty E., El Rakhawy M. (2015). Levonorgestrel-releasing intrauterine system vs oral progestins for non-atypical endometrial hyperplasia: A systematic review and metaanalysis of randomized trials. Am. J. Obstet. Gynecol..

[29] Shen Y., Fang H., Zhang Y., Du Y., Cai R., Zhao M., Chen Q. (2022). Comparison of the effectiveness of the levonorgestrel-intrauterine device and oral progestogens on regression of endometrial hyperplasia without atypia. Heliyon.

[30] Nooh A.M., Abdeldayem H.M., Girbash E.F., Arafa E.M., Atwa K., Abdel-Raouf S.M. (2016). Depo-Provera Versus Norethisterone Acetate in Management of Endometrial Hyperplasia Without Atypia. Reprod. Sci..

[31] Ismail M.T., Fahmy D.M., Elshmaa N.S. (2013). Efficacy of levonorgestrel-releasing intrauterine system versus oral progestins in treatment of simple endometrial hyperplasia without atypia. Reprod. Sci..

[32] Behnamfar F., Ghahiri A., Tavakoli M. (2014). Levonorgestrel-releasing intrauterine system (Mirena) in compare to medroxyprogesterone acetate as a therapy for endometrial hyperplasia. J. Res. Med. Sci..

[33] Orbo A., Vereide A., Arnes M., Pettersen I., Straume B. (2014). Levonorgestrel-impregnated intrauterine device as treatment for endometrial hyperplasia: A national multicentre randomised trial. BJOG Int. J. Obstet. Gynaecol..

[34] Dolapcioglu K., Boz A., Baloglu A. (2013). The efficacy of intrauterine versus oral progestin for the treatment of endometrial hyperplasia. A prospective randomized comparative study. Clin. Exp. Obstet. Gynecol..

[35] Gallos I.D., Krishan P., Shehmar M., Ganesan R., Gupta J.K. (2013). LNG-IUS versus oral progestogen treatment for endometrial hyperplasia: A long-term comparative cohort study. Hum. Reprod..

[36] Lee Y., Miron A., Drapkin R., Nucci M.R., Medeiros F., Saleemuddin A., Garber J., Birch C., Mou H., Gordon R.W. (2007). A candidate precursor to serous carcinoma that originates in the distal fallopian tube. J. Pathol..

[37] Mourits M.J., Bijen C.B., Arts H.J., ter Brugge H.G., van der Sijde R., Paulsen L., Wijma J., Bongers M.Y., Post W.J., van der Zee A.G. (2010). Safety of laparoscopy versus laparotomy in early-stage endometrial cancer: A randomised trial. Lancet Oncol..

[38] Edris F., Vilos G.A., Al-Mubarak A., Ettler H.C., Hollett-Caines J., Abu-Rafea B. (2007). Resectoscopic surgery may be an alternative to hysterectomy in high-risk women with atypical endometrial hyperplasia. J. Minim. Invasive Gynecol..

[39] Vilos G.A., Oraif A., Vilos A.G., Ettler H., Edris F., Abu-Rafea B. (2015). Long-term clinical outcomes following resectoscopic endometrial ablation of non-atypical endometrial hyperplasia in women with abnormal uterine bleeding. J. Minim. Invasive Gynecol..

[40] Scarselli G., Bargelli G., Taddei G.L., Marchionni M., Peruzzi E., Pieralli A., Mattei A., Buccoliero A.M., Fambrini M. (2011). Levonorgestrel-releasing intrauterine system (LNG-IUS) as an effective treatment option for endometrial hyperplasia: A 15-year follow-up study. Fertil. Steril..

[41] Gallos I.D., Ganesan R., Gupta J.K. (2013). Prediction of regression and relapse of endometrial hyperplasia with conservative therapy. Obstet. Gynecol..

[42] Gunderson C.C., Fader A.N., Carson K.A., Bristow R.E. (2012). Oncologic and reproductive outcomes with progestin therapy in women with endometrial hyperplasia and grade 1 adenocarcinoma: A systematic review. Gynecol. Oncol..

[43] Laurelli G., Di Vagno G., Scaffa C., Losito S., Del Giudice M., Greggi S. (2011). Conservative treatment of early endometrial cancer: Preliminary results of a pilot study. Gynecol. Oncol..

[44] Kopatsaris S., Apostolopoulou A., Tsakiridis I., Tranidou A., Zachomitros F., Papanikolaou E., Daponte A., Kalogiannidis I., Dagklis T. (2024). Accuracy of Frozen Section Biopsy in the Diagnosis of Endometrial Cancer: A Systematic Review and Meta-Analysis. Cancers.

[45] Indermaur M.D., Shoup B., Tebes S., Lancaster J.M. (2007). The accuracy of frozen pathology at time of hysterectomy in patients with complex atypical hyperplasia on preoperative biopsy. Am. J. Obstet. Gynecol..

[46] Attard Montalto S., Coutts M., Devaja O., Summers J., Jyothirmayi R., Papadopoulos A. (2008). Accuracy of frozen section diagnosis at surgery in pre- malignant and malignant lesions of the endometrium. Eur. J. Gynaecol. Oncol..

[47] Chaiken S.R., Bohn J.A., Bruegl A.S., Caughey A.B., Munro E.G. (2022). Hysterectomy with a general gynecologist vs gynecologic-oncologist in the setting of endometrial intraepithelial neoplasia: A cost-effectiveness analysis. Am. J. Obstet. Gynecol..

[48] Marcickiewicz J., Sundfeldt K. (2011). Accuracy of intraoperative gross visual assessment of myometrial invasion in endometrial cancer. Acta Obstet. Gynecol. Scand..

[49] Traen K., Hølund B., Mogensen O. (2007). Accuracy of preoperative tumor grade and intraoperative gross examination of myometrial invasion in patients with endometrial cancer. Acta Obstet. Gynecol. Scand..

[50] Trimble C.L., Kauderer J., Zaino R., Silverberg S., Lim P.C., Burke J.J., Alberts D., Curtin J. (2006). Concurrent endometrial carcinoma in women with a biopsy diagnosis of atypical endometrial hyperplasia: A Gynecologic Oncology Group study. Cancer.

[51] Panici P.B., Basile S., Maneschi F., Lissoni A.A., Signorelli M., Scambia G., Angioli R., Tateo S., Mangili G., Katsaros D. (2008). Systematic pelvic lymphadenectomy vs. no lymphadenectomy in early-stage endometrial carcinoma: Randomized clinical trial. J. Natl. Cancer Inst..

[52] Kitchener H., Swart A.M., Qian Q., Amos C., Parmar M.K., ASTEC Study Group (2009). Efficacy of systematic pelvic lymphadenectomy in endometrial cancer (MRC ASTEC trial): A randomised study. Lancet.

[53] Westin S.N., Fellman B., Sun C.C., Broaddus R.R., Woodall M.L., Pal N., Urbauer D.L., Ramondetta L.M., Schmeler K.M., Soliman P.T. (2021). Prospective phase II trial of levonorgestrel intrauterine device: Nonsurgical approach for complex atypical hyperplasia and early-stage endometrial cancer. Am. J. Obstet. Gynecol..

[54] Cholakian D., Hacker K., Fader A.N., Gehrig P.A., Tanner E.J. (2016). Effect of oral versus intrauterine progestins on weight in women undergoing fertility preserving therapy for complex atypical hyperplasia or endometrial cancer. Gynecol. Oncol..

[55] Chen M., Jin Y., Li Y., Bi Y., Shan Y., Pan L. (2016). Oncologic and reproductive outcomes after fertility-sparing management with oral progestin for women with complex endometrial hyperplasia and endometrial cancer. Int. J. Gynecol. Obstet..

[56] Pronin S.M., Novikova O.V., Andreeva J.Y., Novikova E.G. (2015). Fertility-Sparing Treatment of Early Endometrial Cancer and Complex Atypical Hyperplasia in Young Women of Childbearing Potential. Int. J. Gynecol. Cancer.

[57] Ohyagi-Hara C., Sawada K., Aki I., Mabuchi S., Kobayashi E., Ueda Y., Yoshino K., Fujita M., Tsutsui T., Kimura T. (2015). Efficacies and pregnant outcomes of fertility-sparing treatment with medroxyprogesterone acetate for endometrioid adenocarcinoma and complex atypical hyperplasia: Our experience and a review of the literature. Arch. Gynecol. Obstet..

[58] Shan W., Wang C., Zhang Z., Gu C., Ning C., Luo X., Zhou Q., Chen X. (2014). Conservative therapy with metformin plus megestrol acetate for endometrial atypical hyperplasia. J. Gynecol. Oncol..

[59] Mitsuhashi A., Sato Y., Kiyokawa T., Koshizaka M., Hanaoka H., Shozu M. (2016). Phase II study of medroxyprogesterone acetate plus metformin as a fertility-sparing treatment for atypical endometrial hyperplasia and endometrial cancer. Ann. Oncol..

[60] Kopatsaris S., Tsakiridis I., Kapetanios G., Zachomitros F., Michos G., Papanikolaou E., Athanasiadis A., Dagklis T., Kalogiannidis I. (2024). Management of Endometrial Cancer: A Comparative Review of Guidelines. Cancers.

[61] Gallos I.D., Yap J., Rajkhowa M., Luesley D.M., Coomarasamy A., Gupta J.K. (2012). Regression, relapse, and live birth rates with fertility-sparing therapy for endometrial cancer and atypical complex endometrial hyperplasia: A systematic review and metaanalysis. Am. J. Obstet. Gynecol..

[62] Reed S.D., Newton K.M., Garcia R.L., Allison K.H., Voigt L.F., Jordan C.D., Epplein M., Swisher E., Upson K., Ehrlich K.J. (2010). Complex hyperplasia with and without atypia: Clinical outcomes and implications of progestin therapy. Obstet. Gynecol..

[63] Gallos I.D., Krishan P., Shehmar M., Ganesan R., Gupta J.K. (2013). Relapse of endometrial hyperplasia after conservative treatment: A cohort study with long-term follow-up. Hum. Reprod..

[64] Vaugon M., Peigné M., Phelippeau J., Gonthier C., Koskas M. (2021). IVF impact on the risk of recurrence of endometrial adenocarcinoma after fertility-sparing management. Reprod. Biomed. Online.

[65] Bian J., Shao H., Liu H., Li H., Fang L., Xing C., Wang L., Tao M. (2015). Efficacy of the Levonorgestrel-Releasing Intrauterine System on IVF-ET Outcomes in PCOS With Simple Endometrial Hyperplasia. Reprod. Sci..

[66] Mandelbaum R.S., Ciccone M.A., Nusbaum D.J., Khoshchehreh M., Purswani H., Morocco E.B., Smith M.B., Matsuzaki S., Dancz C.E., Ozel B. (2020). Progestin therapy for obese women with complex atypical hyperplasia: Levonorgestrel-releasing intrauterine device vs systemic therapy. Am. J. Obstet. Gynecol..

[67] Trimble C.L., Method M., Leitao M., Lu K., Ioffe O., Hampton M., Higgins R., Zaino R., Mutter G.L. (2012). Management of endometrial precancers. Obstet. Gynecol..

[68] Ørbo A., Arnes M., Vereide A.B., Straume B. (2016). Relapse risk of endometrial hyperplasia after treatment with the levonorgestrel-impregnated intrauterine system or oral progestogens. BJOG Int. J. Obstet. Gynaecol..

[69] Kelly P., Dobbs S.P., McCluggage W.G. (2007). Endometrial hyperplasia involving endometrial polyps: Report of a series and discussion of the significance in an endometrial biopsy specimen. BJOG Int. J. Obstet. Gynaecol..

[70] de Rijk S.R., Steenbergen M.E., Nieboer T.E., Coppus S.F. (2016). Atypical Endometrial Polyps and Concurrent Endometrial Cancer: A Systematic Review. Obstet. Gynecol..

[71] Furness S., Roberts H., Marjoribanks J., Lethaby A. (2012). Hormone therapy in postmenopausal women and risk of endometrial hyperplasia. Cochrane Database Syst. Rev..

[72] Wells M., Sturdee D.W., Barlow D.H., Ulrich L.G., O’Brien K., Campbell M.J., Vessey M.P., Bragg A.J. (2002). Effect on endometrium of long term treatment with continuous combined oestrogen-progestogen replacement therapy: Follow up study. BMJ.

[73] Sturdee D.W., Ulrich L.G., Barlow D.H., Wells M., Campbell M.J., Vessey M.P., Nielsen B., Anderson M.C., Bragg A.J. (2000). The endometrial response to sequential and continuous combined oestrogen-progestogen replacement therapy. BJOG Int. J. Obstet. Gynaecol..

[74] van Leeuwen F.E., Benraadt J., Coebergh J.W., Kiemeney L.A., Gimbrère C.H., Otter R., Schouten L.J., Damhuis R.A., Bontenbal M., Diepenhorst F.W. (1994). Risk of endometrial cancer after tamoxifen treatment of breast cancer. Lancet.

[75] Fisher B., Costantino J.P., Wickerham D.L., Cecchini R.S., Cronin W.M., Robidoux A., Bevers T.B., Kavanah M.T., Atkins J.N., Margolese R.G. (2005). Tamoxifen for the prevention of breast cancer: Current status of the National Surgical Adjuvant Breast and Bowel Project P-1 study. J. Natl. Cancer Inst..

[76] Gibson L., Lawrence D., Dawson C., Bliss J. (2009). Aromatase inhibitors for treatment of advanced breast cancer in postmenopausal women. Cochrane Database Syst. Rev..

[77] Dominick S., Hickey M., Chin J., Su H.I. (2015). Levonorgestrel intrauterine system for endometrial protection in women with breast cancer on adjuvant tamoxifen. Cochrane Database Syst. Rev..

[78] Shi Q., Li J., Li M., Wu J., Yao Q., Xing A. (2014). The role of levonorgestrel-releasing intrauterine system for endometrial protection in women with breast cancer taking tamoxifen. Eur. J. Gynaecol. Oncol..

[79] Wong A.W.Y., Chan S.S.C., Yeo W., Yu M.Y., Tam W.H. (2013). Prophylactic use of levonorgestrel-releasing intrauterine system in women with breast cancer treated with tamoxifen: A randomized controlled trial. Obstet. Gynecol..

[80] Trinh X.B., Tjalma W.A., Makar A.P., Buytaert G., Weyler J., van Dam P.A. (2008). Use of the levonorgestrel-releasing intrauterine system in breast cancer patients. Fertil. Steril..

